# In-Plane Behavior of Auxetic Non-Woven Fabric Based on Rotating Square Unit Geometry under Tensile Load

**DOI:** 10.3390/polym11061040

**Published:** 2019-06-12

**Authors:** Polona Dobnik Dubrovski, Nejc Novak, Matej Borovinšek, Matej Vesenjak, Zoran Ren

**Affiliations:** 1 Institute of Engineering Materials and Design, Faculty of Mechanical Engineering, University of Maribor, Maribor 2000, Slovenia; 2 Mechanical Engineering Research Institute, Faculty of Mechanical Engineering, University of Maribor, Maribor 2000, Slovenia; n.novak@um.si (N.N.); matej.borovinsek@um.si (M.B.); matej.vesenjak@um.si (M.V.); zoran.ren@um.si (Z.R.)

**Keywords:** auxetic polymer materials, needle-punched non-woven fabric, rotating square unit geometry, mechanical properties, tensile load, Poisson’s ratio

## Abstract

This paper reports the auxetic behavior of modified conventional non-woven fabric. The auxetic behavior of fabric was achieved by forming rotating square unit geometry with a highly ordered pattern of slits by laser cutting. Two commercial needle-punched non-woven fabric used as lining and the reinforcement fabric for the footwear industry were investigated. The influence of two rotating square unit sizes was analyzed for each fabric. The original and modified fabric samples were subjected to quasi-static tensile load by using the Tinius Olsen testing machine to observe the in-plane mechanical properties and deformation behavior of tested samples. The tests were recorded with a full high-definition (HD) digital camera and the video recognition technique was applied to determine the Poisson’s ratio evolution during testing. The results show that the modified samples exhibit a much lower breaking force due to induced slits, which in turn limits the application of such modified fabric to low tensile loads. The samples with smaller rotating cell sizes exhibit the highest negative Poisson’s ratio during tensile loading through the entire longitudinal strain range until rupture. Non-woven fabric with equal distribution and orientation of fibers in both directions offer better auxetic response with a smaller out-of-plane rotation of rotating unit cells. The out-of-plane rotation of unit cells in non-homogenous samples is higher in machine direction.

## 1. Introduction

**Figure 1:**
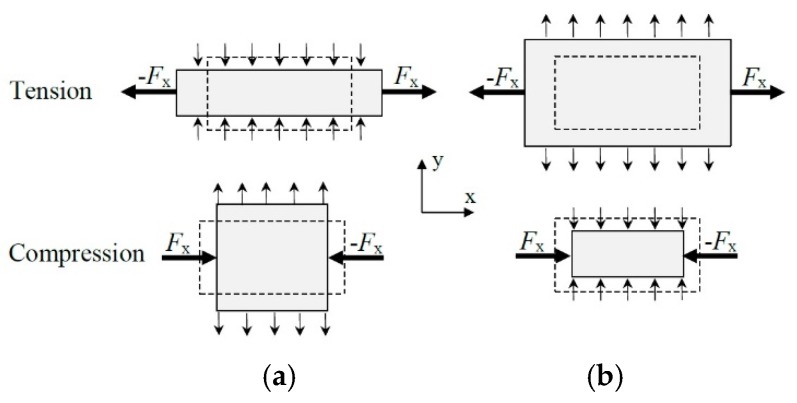
Non-auxetic (**a**) and auxetic (**b**) behavior during tensile and compressive loading (dashed lines—undeformed geometry)

Textiles are natural or synthetic polymer materials that are manufactured in the form of fibers, yarns or fabric and are used for clothing, interior design and many different technical applications. Conventional textile materials have a positive Poisson’s ratio (ranging from 0.0 to 0.5). The auxetic materials exhibit a negative Poisson’s ratio, which means that, under tension, they elongate both in the direction of loading and in the transversal direction and vice versa under compression loading, Figure 1 [1,2,3]. 

In the last few decades, research on auxetic textile materials has been focused on developing some enhanced properties such as the ability to form dome-shaped structures (synclastic curvature) when subjected to a bending load, indentation resistance, good vibration damping and shock absorption, ability to manage porosity and air permeability (under pressure), enhanced acoustic properties, lower stiffness, low density, higher formability, better compatibly with the body, size fitting, etc. [4,5,6,7]. Such materials have their potentials as technical textiles in the medicine, automobile, marine and aerospace, architecture, civil engineering, and footwear industries, etc., for filtration where the pore opening size can be controlled by tension, vibration damping, shock absorbency, smart bandages, dental floss with in-built drug release, compression hosiery, seat cushion material, fastening devices, reinforcements in advanced composites, padding material for better forefoot pressure relief in high-heeled shoes, as well as apparel textiles (maternity dresses, bra cups, leggings, children’s wear during the period of growth, support garments, etc.) [4,5,6,7,8,9,10,11,12,13,14]. 

The auxetic properties of textiles can be reached at fiber, yarn and fabric levels by changing their structure. Comprehensive reviews of auxetic fibers, yarns and fabric are given in the literature [12,15,16,17,18]. There are two possible ways to induce auxetic properties in two-dimensional (2D) fabric:

- by using auxetic fibers/yarns in the conventional process of weaving, knitting, braiding and non-woven production, or
- by inducing special (auxetic) structures (geometry) in the conventional process of weaving, knitting, and non-woven production using conventional fibers/yarns.

The latter way to induce auxetic fabric offers low cost and continuous usage of conventional manufacturing equipment. Some auxetic 2D geometries have already been developed using traditional textile technologies:

- re-entrant geometry (hexagonal geometry in the case of knitted and woven fabric [5,6,18], double arrowhead [19] and rhombus-shaped geometry [16] in the case of warp knitted fabric);
- rotating square unit geometry for knitted fabric [5];
- foldable geometry for knitted and woven fabric [4,5,12,20].

Auxetic re-entrant geometry has also been developed by combining three-dimensional (3D) printing with traditional weft knitting technology to form a multi-material system with enhanced mechanical (auxetic behavior) and porous properties [21]. 

The majority of research is aimed at the development and prediction of the in-plane auxetic behavior of knitted and woven auxetic fabric, while there is a substantial lack in development of the auxetic non-woven fabric, as well as a lack of predicting the out-of-plane behavior, which is completely ignored, despite the fact that auxetic materials do exist in 3D form [15]. The auxetic composite’s out-of-plane behavior was analyzed in [22], where it was shown that the out-of-plane auxeticity of the composite is dependent on the in-plane properties of the honeycomb. Verma et al. [23] reported that a heat compression protocol used on needle-punched commercial non-woven fabric induces an out-of-plane auxetic response. More precisely, tested heat-compressed needle-punched non-woven fabric have shown an increase in thickness when stretched, especially at strains lower than 30%. The observed Poisson’s ratio at 5% strain was −7.2 and −6.6 for two tested samples, respectively. Bhullar et al. [24] developed non-woven fabric, where auxetic geometry was tailored using laser micromachining on a polycaprolactone microfiber and a polycaprolactone sheet.

**Figure 2:**
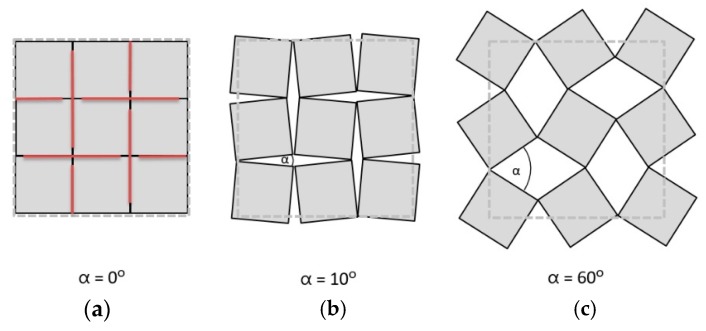
The geometry of auxetic rotating squares structures: (**a**) undeformed structure and (**b**,**c**) deformed structures.

The auxetic behavior can be also achieved by the material’s internal structure geometry, which changes as a mechanism under applied loads. Grima et al. [25,26,27] proposed rotating rigid unit cells in the form of squares, triangles and rectangles, connected together at selected vertices by hinges (Figure 2). 

This paper reports on a study of using needle-punched technology and laser cutting (in order to form the geometry of rotating squares) for fabricating auxetic non-woven fabric. The comparison analysis between non-auxetic and auxetic non-woven fabric behavior under quasi-static tensile load and the determination of Poisson’s ratio are demonstrated and discussed. 

## 2. Materials and Methods 

The simplest way to induce the in-plane auxetic behavior of conventional needle-punched non-woven fabric is to form rotating unit cells with a highly ordered pattern of slits by using laser cutting. The needle-punched non-woven fabric investigated in this study was a commercial Silon fabric (obtained from Konus-Konex, Slov. Konjice, Slovenia), which is a synthetic leather used for heel grip, insole and lining material (in the footwear industry), as well as lining material in the manufacturing of belts. The idea to use auxetic material for inner parts of shoes lies in the possibility to design shoes, which will be able to enlarge their size in the case of swollen feet. In this case, it is not only the outer fabric that should have auxetic properties, but also the inner parts of the shoe. Two needle-punched non-woven fabric, referred to as SL-1 and SL-2 from here on, were investigated. The basic structural characteristics of the tested fabric are given in Table 1. As mentioned above, Silon fabric is a non-woven fabric made by a conventional procedure of web forming on a card line, which is then reinforced on the basis of needle-punching technology and finally finished using splitting and buffing techniques.

The geometry of the applied rotating square unit cells of the same pattern is given in Table 2. The influence of the two rotating square unit cell sizes, e.g., 1.25 × 1.25 cm and 0.625 cm × 0.625 cm, connected at selected vertices by 2-mm long hinges was investigated for each tested fabric.

It should be mentioned that the two different geometries of the rotating unit cells involved in this study (bigger and smaller) are not scaled versions. The thickness of the slits and the size of the hinges remained the same for the small unit cells to avoid premature failure in case of too week hinges.

Fifteen test samples of each needle-punched non-woven fabric were first cut in the machine and fifteen in the cross-machine direction, with the overall dimensions of 50 ± 0.5 mm × 250 ± 0.5 mm (width × length). The machine direction means the direction of fabric forming, e.g., the length of fabric roll, while the cross-machine direction means the width of the fabric roll. Five plus five samples of each needle-punched non-woven fabric were then modified by inducing the pattern of slits by laser cutting to form rotating unit cells of two sizes. The samples were then subjected to the conditioning for 48 hours before testing. 

Quasi-static tensile measurements were performed using the Tinius Olsen testing machine H10KT (Tinius Olsen Ltd., Redhill, United Kingdom) with flat-faced clamps and a 1000-N load cell, following the ISO EN 9073 standard. The conditions at tensile testing were as follows: gauge length—150 mm, constant rate of extension—100 mm/min, and standard atmosphere. The maximum breaking force and elongation at break were recorded for all samples, and then the average values were calculated, expressed in N/5 cm and %, respectively.

To determine whether there were any differences among the samples according to the breaking force and elongation at break regarding the type of material, the direction of the material taken from the roll fabric and the type of geometry, and to test the null hypotheses (there are no differences between the groups regarding the upper mentioned factors), analysis of variance procedure (ANOVA) was performed using IBM SPSS 22 statistical software package (IBM Corporation, New York, NY, United States). The selected value of significance level for this procedure was 0.05 (or 95% confidence level). 

The Poisson’s ratio of all tested samples was determined by using a video image recognition methodology as an engineering value. A video analysis software based on Accord.NET framework for scientific computing was developed for that purpose. The width of the samples was measured in time, which was determined from the video frame rate. The sample location on the video was determined for each video image (frame) by using a template matching object tracker of the movable clamp of the testing machine. The image filtering was used to segment the samples from the background based on a Canny edge detector [28]. The width of the sample was then measured in pixels from the segmented image and converted to the transversal strain. The time-dependent evolution of the Poisson’s ratio was finally computed as the ratio of the measured transversal and longitudinal strain.

## 3. Results and Discussion

### 3.1. Fabric Structure Analysis

**Figure 3:**
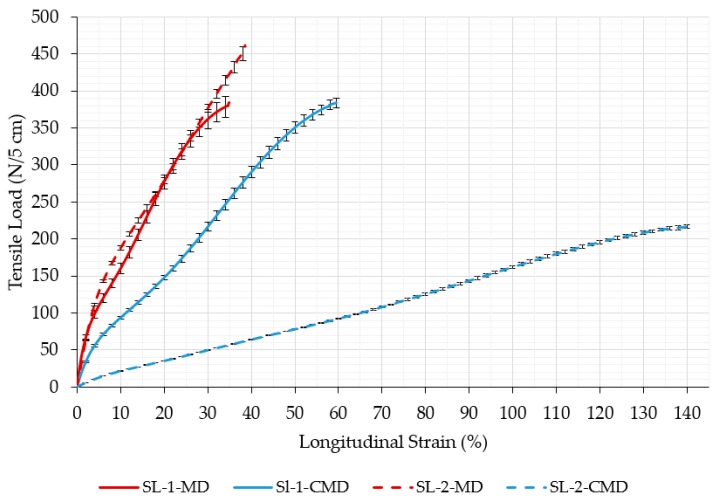
Tensile behavior of the original non-woven Silon (SL) fabric. (MD—machine direction; CMD—cross-machine direction).

Figure 3 shows the tensile strength relationships of both original (non-auxetic) non-woven fabric in the machine (MD) and cross-machine directions (CMD), while the maximum values are listed in Table 3. 

The SL-1 fabric is obviously more homogenous since it exhibits comparable properties in machine and cross-machine directions, while the properties of sample SL-2 are quite different. The SL-1 fabric has approximately the same breaking force in machine and cross-machine directions, while the elongation at break is higher in the cross-machine direction. This implies that fiber orientation is a little lower in this direction. The fibers in SL-2 fabric are obviously more heterogeneously distributed since the tensile strength in the machine direction is over 2 times higher than in the cross-machine direction, while the elongation at break is almost 3.5 times larger in the cross-machine direction in comparison to the machine direction. 

### 3.2. Auxetic Behaviour Analysis

**Figure 4:**
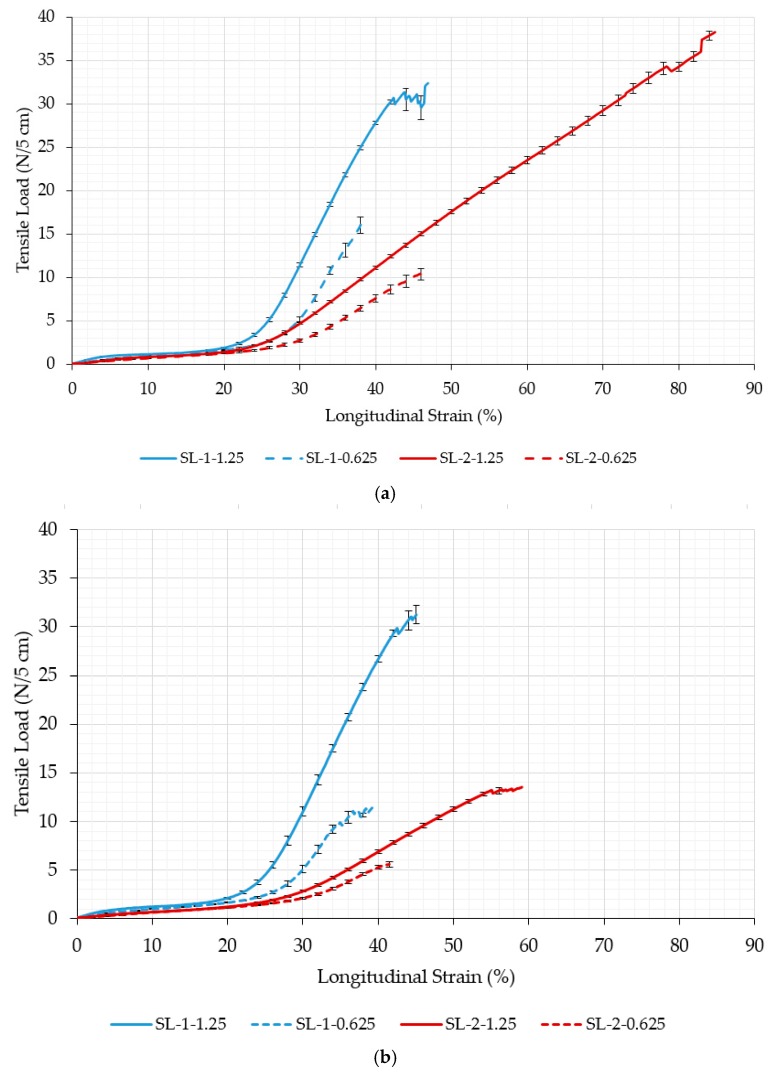
Tensile behavior of auxetic fabric in the (**a**) machine and (**b**) cross-machine directions.

The comparison between two different auxetic geometries regarding the behavior of auxetic non-woven fabric (ANF) under tensile load is presented in the form of tensile strength relationships for the machine and cross-machine directions in Figure 4. The results show that auxetic samples with a larger unit cell size (1.25 cm) break at higher force and elongation in comparison to the auxetic samples with a smaller unit cell size (0.625 cm). Again, sample SL-1 exhibits comparable properties in the machine and cross-machine directions; while sample SL-2 shows much higher values of breaking strength and elongation in the machine direction in comparison to the cross-machine direction. 

### 3.3. Breaking Strength and Elongation Analysis

Table 3 shows the average values with standard deviations of breaking strength and elongation at break for the non-auxetic (NF) and auxetic non-woven (ANF) tested fabric in the machine and cross-machine directions. 

The results of the ANOVA analysis (Table 4 and Table 5) show that there are statistically significant differences between the groups of samples regarding the type of material (SL-1 and SL-2), the direction of material taken from the roll (MD and CMD) and the type of geometry (samples with a rotating unit size of 1.25 cm and samples with a rotating unit size of 0.625 cm); the value of significance level is lower than 0.001, so all these factors have a statistically significant effect on the breaking force and elongation at break of the tested samples.

The results show a large reduction in breaking force due to laser cutting, which was used to introduce the auxetic geometry into the fabric. The reduction in tensile strength in samples with a rotating unit size of 1.25 cm is approx. 94 and 92% in comparison to the original tensile strength in the machine direction for samples SL-1 and SL-2, respectively. The reason for the lower breaking force can be attributed to the massive reduction in the specimen cross-section area due to the induced cuts. Slightly larger is the reduction in the tensile strength for samples with a rotating unit size of 0.625 cm (96 and 98% for SL-1 and SL-2 fabric, respectively). The average reduction in the original tensile strength in the cross-machine direction is approx. 95% for both samples.

The analysis of elongation at break due to the introduction of auxetic geometry shows different behavior regarding the testing direction and homogeneity of fiber orientation in both directions. On inducing auxetic geometry, the elongation at break increases in the machine direction, while it decreases in the cross-machine direction. SL-1 and SL-2 fabric show the same behavior in machine direction: on inducing auxetic geometry, the breaking elongation is increased by approx. 70 or 11% for samples with a rotating unit size of 1.25 and 0.625 cm, respectively. Here, the SL-2 fabric, which is less homogenous, shows a much higher increase in elongation in comparison with the SL-1 fabric. In the cross-machine direction, the SL-1 and SL-2 fabric again show similar (albeit opposite) behavior: on inducing auxetic geometry, the elongation at break is reduced by approx. 41 or 52% for samples with a rotating unit size of 1.25 and 0.625 cm, respectively. Here, the SL-2 fabric, which is less homogenous, again shows a much higher decrease in elongation at break in comparison with SL-1 fabric.

### 3.4. Poisson’s Ratio Evaluation

**Figure 5:**
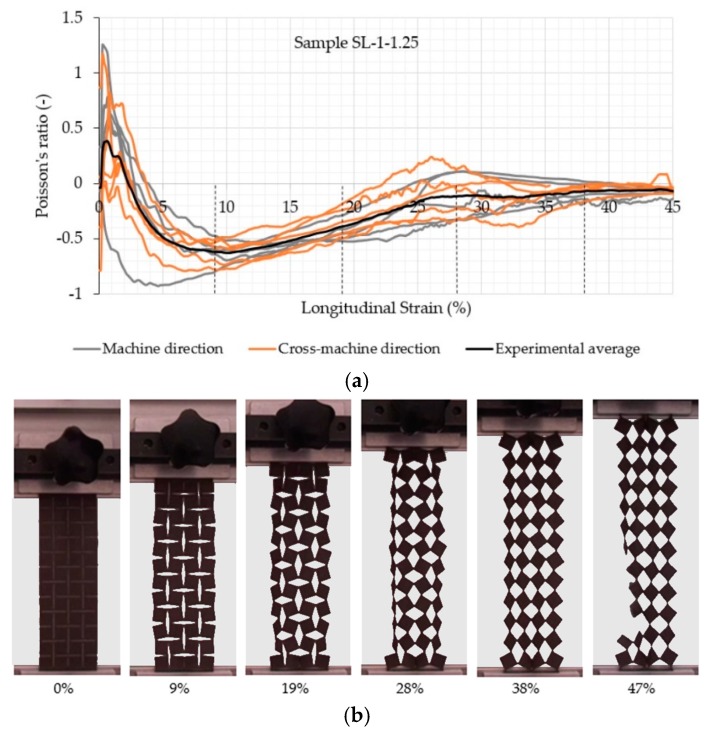
(**a**) Evolution of the Poisson’s ratio with the longitudinal strain of the auxetic sample SL-1-1.25 and (**b**) fabric deformation at different longitudinal strains during tensile testing in the machine direction.

**Figure 6:**
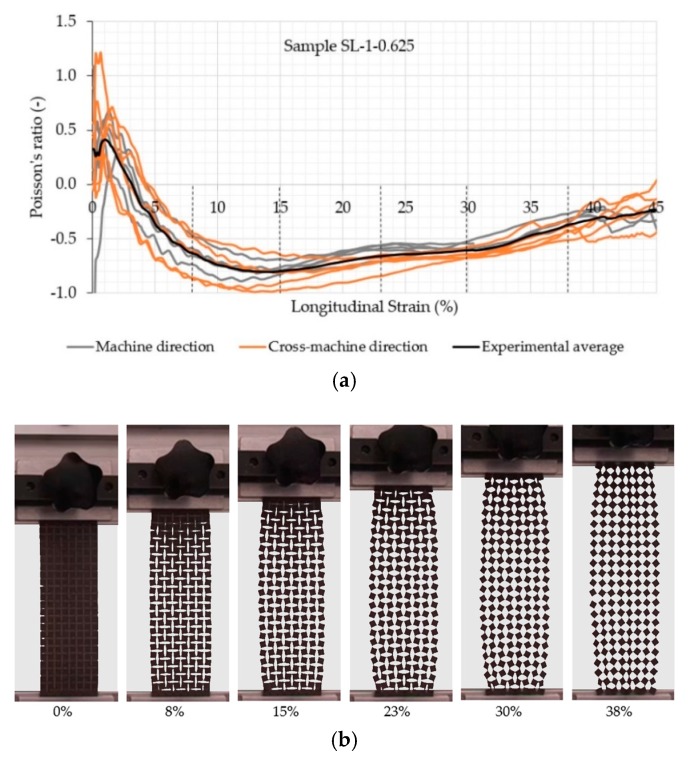
(**a**) Evolution of the Poisson’s ratio with the longitudinal strain of the auxetic sample SL-1-0.625 and (**b**) fabric deformation at different longitudinal strains during tensile testing in the machine direction.

**Figure 7:**
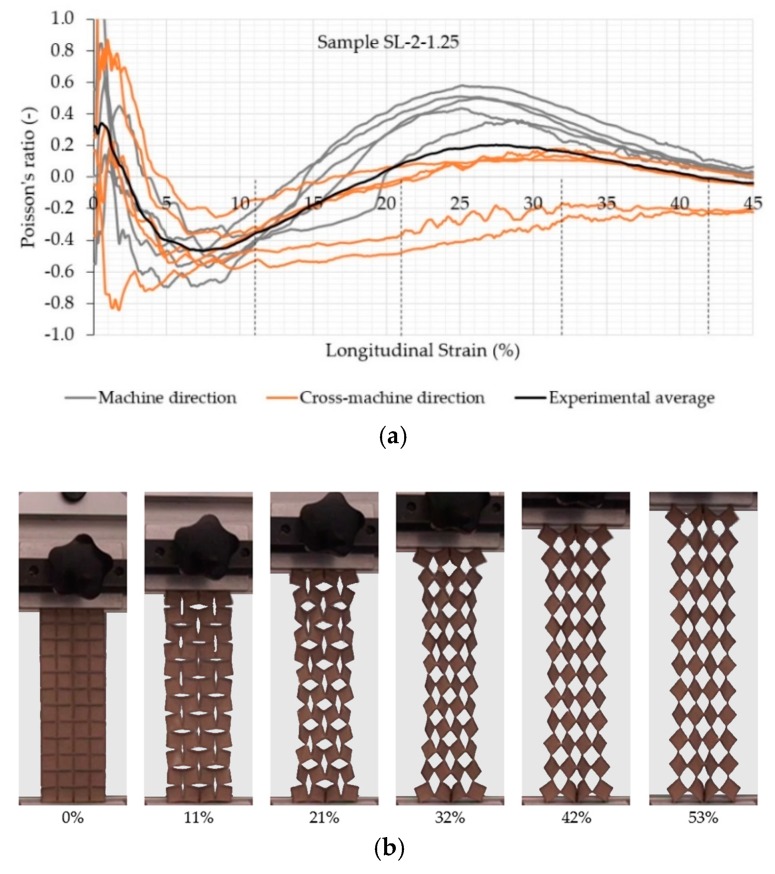
(**a**) Evolution of the Poisson’s ratio with the longitudinal strain of the auxetic sample SL-2-1.25 and (**b**) fabric deformation at different longitudinal strains during tensile testing in the machine direction.

**Figure 8:**
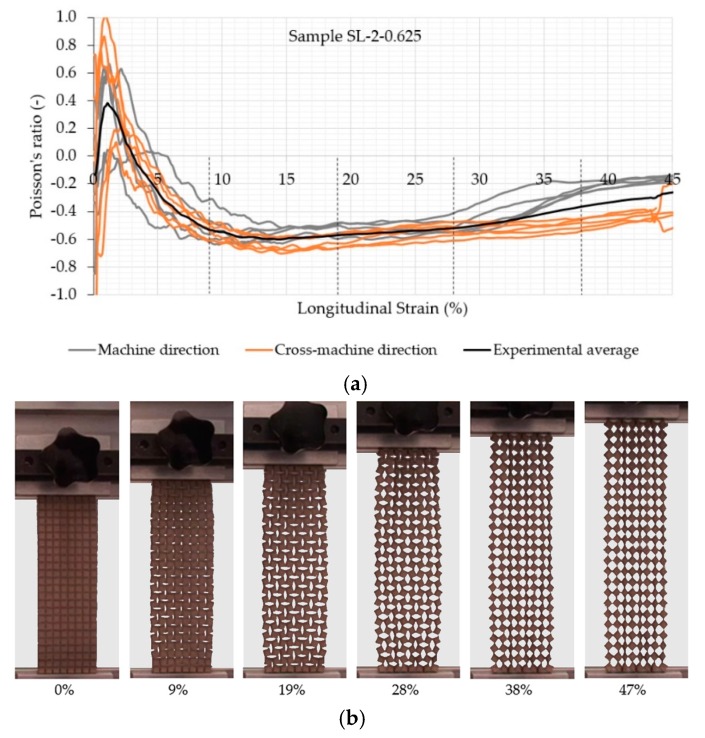
(**a**) Evolution of the Poisson’s ratio with the longitudinal strain of the auxetic sample SL-2-0.625 and (**b**) fabric deformation at different longitudinal strains during tensile testing in the machine direction.

A clear auxetic behavior of the samples can be observed from Figure 5, Figure 6, Figure 7 and Figure 8, which represents the relationship between the Poisson’s ratio and longitudinal strain for the individual samples and corresponding fabric deformation at different longitudinal strains during tensile testing in the machine direction. All relationships show a positive Poisson’s ratio at low longitudinal strains, which is a consequence of the initial alignment of the samples, clamped into the upper and lower clamps without preloading. By increasing the longitudinal strain, the square unit cells start to rotate in plane around the hinges, thus inducing the overall in-plane auxetic behavior. Some out-of-plane unit cell rotation was observed at high longitudinal strains, causing a decrease in the Poisson’s ratio.

A comparison of auxetic behavior between two different geometries of SL-1 fabric (SL-1-1.25 and SL-1-0.625; see experimental average relationships in Figure 5 and Figure 6) shows an obvious difference: the geometry with a rotating cell unit size of 0.625 cm exhibits a larger negative Poisson’s ratio (NPR) across the entire range of the longitudinal strain. This means that SL-1-0.625 samples expand more in the lateral direction. It was observed during testing that the rotation of SL-1-0.625 unit cells occurred only in-plane, contributing to a larger lateral extension, while SL-1-1.25 unit cells also started to rotate out-of-plane at strains larger than 15%. This phenomenon reduces lateral extension and eventually even leads to a positive Poisson’s ratio (see the range of the longitudinal strains between 20 and 40% and fabric deformation at 28% of the longitudinal strain in Figure 5). The highest average NPR is achieved at approx. 10% (−0.6) and 14% (−0.8) of the longitudinal strain for geometry with a 1.25 and 0.625 cm rotating unit size, respectively, regardless of the testing direction. It is also worth mentioning that samples with a rotating unit size of 0.625 cm exhibit auxetic behavior (negative Poisson’s ratio) until the rupture of the samples. From the images in Figure 5 and Figure 6, it can be observed that near the breaking point, the slits are being deformed in such a way that they form an empty unit cell of the same size as the rotating unit cell. The large increase in the stiffness can be noted in the region of 20–30% of the longitudinal strain from Figure 4, i.e., a higher force is needed for deformation. From Figure 7b and Figure 8b, it can be observed that the squares rotate up to 20–30% of the longitudinal strain. Here, it seems that the deformation is mainly caused by the structural properties of the samples, i.e., the auxetic patterns of cuts, whereas above 30% of the longitudinal strain (rotating units reach a rotation angle of 45°), the rotation mechanism cannot support further deformation. Therefore, the deformation is mainly influenced by the mechanical properties of the connections between the rotating units, e.g., the mechanical properties of the material itself. Here, a much higher force is needed for deformation, until the connections between the rotating units start to break and a maximum breaking force is detected—see Figure 5 and Figure 6.

The comparison of auxetic behavior between two different geometries of SL-2 (SL-2-1.25 and SL-2-0.625; see experimental average relationships in Figure 7 and Figure 8) also shows an obvious difference: the geometry with a rotating cell size 0.625 cm exhibits a much higher NPR across the complete range of the longitudinal strain. The highest average NPR is achieved at approx. 7% (−0.5) and 14% (−0.6) of the longitudinal strain for geometry with a rotating unit size of 1.25 and 0.625 cm. respectively, regardless of the testing direction. Both geometries also show differences in the NPR in both directions due to different fabric homogeneity in the machine and cross-machine direction. In the case of SL-2-0.625, the difference becomes obvious above 30% of the longitudinal strain, where the NPR in the cross-machine direction is higher in comparison with the machine direction, while in the case of SL-2-1.25, the difference is already obvious above 15% of the longitudinal strain. Here, even the Poisson’s ratio is positive for machine direction, while the average Poisson’s ratio for the cross-machine direction is still negative or near-zero. During the tensile testing of SL-2-1.25, it was observed that the auxetic units also started to rotate in the out-of-plane direction (normal to the sample surface) at approximately 38% of the longitudinal strain, thus reducing the lateral extension of the sample in the machine direction—see Figure 7. The out-of-plane rotation of cell units was not observed in SL-2-0.625—see Figure 8.

From the results, it is obvious that fiber orientation in the fabric and the geometry of the induced auxetic structure both have an important influence on the auxetic behavior of non-woven fabric.

## 4. Conclusions

The main conclusions from the auxetic behavior analysis of non-woven fabric with two different rotating unit geometries are the following:

- laser cutting, which was used to induce auxetic geometry into non-woven fabric, causes a significant reduction in breaking force; therefore, their application is restricted to low tensile loads;
- tested non-woven samples with induced rotating unit cell geometry with a rotating unit size of 0.625 cm exhibit a higher negative Poisson’s ratio (up to −1.0) during tensile loading through the entire longitudinal strain range until rupture;
- non-woven fabric with equal distribution and orientation of fibers offer a better auxetic response with a smaller out-of-plane rotation of unit cells;
- the out-of-plane rotation of unit cells in non-homogenous fabric is higher in the machine direction.

This study has shown that auxetic behavior could be induced in conventional textile materials (non-woven fabric) by forming rotating unit cells with a highly ordered pattern of slits by using laser cutting. In this way, the textile materials can be transformed into more value-added metamaterials with a negative Poisson’s ratio. However, there is still a need to further explore the possibilities regarding different loading conditions, geometries in the form of oriented patterns of slits, as well as quasi-random patterns, which do not contain elements of symmetry.

## Figures and Tables

**Table 1:** Structural characteristics of tested non-woven fabric.

Sample ID	Raw Material	Fabric Mass per Unit Area (g·m^−2^)	Fabric Thickness (mm)	Fabric Density (g·cm^−3^)
SL-1	PET fibers	29	0.7	0.041
SL-2	PET fibers	38	0.7	0.054

**Table 2:** Rotating square unit cell geometry.

One Repeat Unit of Rotating Squares Geometry (4 Cells)	Cell Size (cm)	A (mm)	B (mm)	t (mm)
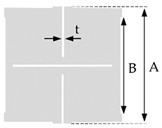	1.25	25	23	0.2
	0.625	12.5	10.5	0.2

**Table 3:** Measurement results of tensile strength and elongation at break.

		Machine Direction (MD)		Cross-Machine Direction (CMD)	
Type of Fabric	Sample Designation	Breaking Force (N/5 cm)	Elongation at Break (%)	Breaking Force (N/5 cm)	Elongation at Break (%)
NF *	**SL1**	384.3 ± 27.9	36.8 ± 1.7	385.1 ± 9.3	59.7 ± 2.0
ANF **	**SL-1-1.25**	32.4 ± 1.4	46.9 ± 1.5	31.2 ± 2.4	45.1 ± 2.3
**ANF**	**SL-1-0.625**	16.3 ± 2.4	38.3 ± 1.0	11.6 ± 0.7	39.5 ± 1.1
**NF**	**SL-2**	459.1 ± 35.6	39.7 ± 2.6	217.0 ± 4.9	139.7 ± 4.6
**ANF**	**SL-2-1.25**	38.3 ± 3.4	84.8 ± 4.4	13.5 ± 0.2	59.1 ± 1.1
**ANF**	**SL-2-0.625**	10.6 ± 1.6	46.7 ± 2.0	5.6 ± 0.7	41.5 ± 1.1

* NF non-woven fabric; ** ANF auxetic non-woven fabric.

**Table 4:** Results of the ANOVA analysis for breaking force.

Source	Type III Sum of Squares	df	Mean Square	F	Sig.
Corrected Model	1,639,050.964 *	11	149,004.633	859.634	0.001
Intercept	1,030,951.149	1	1,030,951.149	5947.739	0.001
Material	5467.609	1	5467.609	31.544	0.001
Geometry	1,483,019.484	2	741,509.742	4277.900	0.001
Direction	30,602.714	1	30,602.714	176.552	0.001
Material & Geometry	5274.742	2	2637.371	15.215	0.001
Material & Direction	28,392.401	1	28,392.401	163.801	0.001
Geometry & Direction	39,859.432	2	19,929.716	114.978	0.001
Material & Geometry & Direction	42,729.241	2	21,364.621	123.256	0.001
Error	7973.409	46	173.335		
Total	2,650,547.505	58			
Corrected Total	1,647,024.373	57			

* R Squared = 0.995 (Adjusted R Squared = 0.994).

**Table 5:** Results of the ANOVA analysis for elongation at break.

Source	Type III Sum of Squares	df	Mean Square	F	Sig.
Corrected Model	47,005.572 *	11	4273.234	713.477	0.001
Intercept	187,114.877	1	187,114.877	31,241.474	0.001
Material	8686.778	1	8686.778	1450.380	0.001
Geometry	6792.977	2	3396.488	567.092	0.001
Direction	3143.862	1	3143.862	524.912	0.001
Material & Geometry	3004.780	2	1502.390	250.845	0.001
Material & Direction	836.336	1	836.336	139.638	0.001
Geometry & Direction	15,760.595	2	7880.297	1315.727	0.001
Material & Geometry & Direction	7093.208	2	3546.604	592.156	0.001
Error	275.508	46	5.989		
Total	237,876.501	58			
Corrected Total	47,281.080	57			

* R Squared = 0.994 (Adjusted R Squared = 0.993).
